# Magnocellular Based Visual Motion Training Improves Reading in Persian

**DOI:** 10.1038/s41598-018-37753-7

**Published:** 2019-02-04

**Authors:** Leila Ebrahimi, Hamidreza Pouretemad, Ali Khatibi, John Stein

**Affiliations:** 1 Institute for Cognitive & Brain Sciences, Shahid Beheshti University, Tehran, Iran; 2 Department of Psychology, Shahid Beheshti University, Tehran, Iran; 3 Department of Psychology, Bilkent University, 06800 Ankara, Turkey; 4 Tehran-Oxford Neurodevelopmental Center, Shahid Beheshti University, Tehran, Iran; 5 Department of Physiology, Anatomy & Genetics, Oxford University, Oxford, UK

## Abstract

The visual magnocellular system is thought to play a crucial role in learning to read. Here therefore, we examined whether magnocellular based training could improve reading in children with visual reading problems. The participants were 24 male primary school students aged between 9–11 (Mean = 9.76, SD = 0.59) with specific reading difficulty. Experimental and control groups were matched for age, sex, educational level, IQ, reading abilities (measured by APRA), magnocellular performance as assessed by a random dot kinematogram (RDK) paradigm and recordings of their saccadic eye movements. The experimental group received twelve magnocellular based visual motion training sessions, twice a week over 6 weeks. During the same period, the control group played a video game with the help of a practitioner. All measures were made just prior to the training and were repeated at the 6^th^, 12^th^ training session and one month later. The experimental group showed significant improvements in magnocellular function, visual errors and reading accuracy during the course of intervention. Follow-up assessment confirmed that these effects persisted one month later. Impaired magnocellular functioning appeared to be an important cause of poor reading in Persian. Hence magnocellular based training could help many children with specific reading difficulties. Also testing magnocellular function could be used as screening tool for detecting dyslexia before a child begins to fail at school.

## Introduction

Specific reading disorder, commonly referred to as “developmental dyslexia”, is the most prevalent learning disability (DSM V, 2013)^1^, and affects 4–9% of school age children^2,3^. It is characterized by a severe difficulty in learning to read despite adequate intelligence, motivation and educational opportunities, and in the absence of clinically defined neurological or sensory deficits^1^. Although a growing body of studies has attempted to determine the underlying mechanisms of the disorder, there is as yet no agreement. However, a considerable number of these efforts have implicated the visual magnocellular system^4,5^.

The visual magnocellular pathway originates in the magnocellular ganglion cells in the retina and projects via the magnocellular layers of the Lateral Geniculate Nucleus (LGN) to the primary visual cortex (V1) situated at the back of the occipital lobe^6^. Two main pathways then project forwards towards the front of the brain. The dorsal one of these is often called the ‘where’ stream; it receives 90% of its visual input from the magnocellular system and projects to the visual motion sensitive area (V5/MT) and thence to the Posterior Parietal Cortex (PPC). This pathway therefore mediates motion perception and object localization^7^ and it plays a major role in directing visual attention and in eye movement control^8^. All of these functions are vital for reading.

Accumulating evidence has demonstrated that this magnocellular system is poorly developed in many dyslexics^9–11^. In the earliest study to show this, Livingstone, Rosen, Drislane and Galaburda (1991) found in post mortem histology that the magnocellular cells in the LGN were significantly smaller and more disorganized in dyslexic brains than in age matched controls^9^. In a more recent study, Giraldo-Chica, Hegarty and Schneider (2015) used high-resolution proton-density weighted MRI scans to directly image the LGN in dyslexic and non-dyslexic individuals^12^. They found that left LGN was significantly smaller and differed in shape in dyslexic individuals, suggesting that they contained thinner magnocellular layers in the left hemisphere. Such abnormal lateralization in dyslexia has been demonstrated many times before^13^. Gori, Seitz, Ronconi, Franceschini and Facoetti (2016) described multiple causal links between magnocellular/dorsal pathway deficits and developmental dyslexia. Their results showed that motion perception, assessed by Motion Dot Coherence (CDM), is impaired in dyslexic children in comparison to both age and reading level matched control groups and that it also predicts future reading skills^14^. Also a magnocellular pathway deficit has been found in dyslexia in logographic languages such as Chinese^11,15^. Genetic studies have shown a relationship between a DCDC-2 Intron 2 deletion, which is a known risk factor for dyslexia^16,17^ and magnocellular function in both normal and dyslexic readers^18,19^.

Measuring coherent motion sensitivity is a well-known and sensitive procedure for assessing magnocellular functioning, and many dyslexic individuals show reduced coherent motion sensitivity compared to matched control groups^4,20–25^. Boets and Cornelissen (2011) showed that students who were diagnosed with dyslexia in third grade, had reduced motion detection sensitivity in kindergarten^26^. Dyslexics demonstrate abnormalities in many other indices of magnocellular dysfunction, such as inaccurate saccades, poor vergence control and longer fixations^27–29^.

These converging findings imply that therapeutic interventions designed to improve magnocellular function may help to alleviate visual reading difficulties. This hypothesis has been tested in several studies. Recently, Lawton (2016) and Lawton and Shelley-Tremblay (2017) investigated the efficacy of figure-ground Discrimination Training on reading ability in individuals with dyslexia^30,31^. This task activates the magnocellular pathway selectively and it enhanced their reading fluency; this was accompanied by improvements in their visual timing deficits, attention, phonological processing and working memory. While these authors used patterns activating magnocellular relative to parvocellular systems, other studies attempted training at the level of MT/V5. Coherent motion is discriminated at these higher visual levels that are known to be dominated by magnocellular input^32^. Chouake, Levy, Javitt and Lavidor (2012), showed that training the magnocellular pathway by detecting progressively faster movements could improve lexical decision and reading accuracy^32^. In another study, a magnocellular based visual-motor intervention was conducted on Chinese children with dyslexia. This intervention consisted of coherent motion detection, visual search, visual tracking and juggling. The results demonstrated that magnocellular pathway function in individuals with dyslexia improved greatly and this was associated with significant increases in phonological awareness^11^. Heth and Lavidor (2015) used tDCS over MT/V5 in adults with dyslexia. The results indicated that active, not sham stimulation, improved reading speed and fluency^33^.

In another kind of study, eye movement training was administered to improve reading skills. Leong *et al*. (2014) gave elementary students 18 sessions of treatment with the King-Devick saccadic training software after which they improved their reading fluency significantly^34^. Dodic *et al*. (2016) used the same software in a cross-over design study and found that the treatment group significantly improved in reading comprehension and reading fluency^35^. This had previously been shown by Sabet *et al*. (2013) for a quite different language system, Persian. She showed that eye movement training improved accommodation facility, and this was associated with increments in comprehension and decrements in reading errors^36^.

At least two conclusions can be drawn from the afore-mentioned findings:

1. Magnocellular functioning clearly plays an important role in reading; achieving even a small improvement has immediate effects.
2. These causal effects are not confined to alphabetic irregular languages like English. They have been demonstrated over the whole continuum of transparent - opaque, alphabetic - logographic scripts^11,20,36,37^. Probably therefore, the greater the improvement in magnocellular function, the greater would be the effects on reading difficulties in any language system. This hypothesis was examined in the current study. Persian speaking children with reading difficulties carried out several computerized and manual tasks designed to improve their magnocellular function. Their reading abilities were compared with those of a matched control group pre and post intervention.

The Persian script, Farsi, is highly opaque^2^. In written Farsi, letters are attached to each other to form a word and the writing direction is from right to left. Also, within sentences, the subject comes at the beginning and the verb comes at the end. Thus, accurate and consistent saccadic eye movements are even more important for the comprehension of a sentence in Farsi.

## Results

### Demographics

Independent t-tests were performed at pre-test. There were no significant differences between experimental and control groups on RDK (t(22) = 0.47, *p* = 0.64), saccadic eye movements (t(22) = −1.54, *p* = 0.13), reading accuracy (t(22) = 0.74, *p* = 0.46), reading comprehension (t(22) = −0.34, *p* = 0.73) or mean number of visual errors (t(22) = −0.39, *p* = 0.69). Both groups performed well below that expected of typical readers of the same age.

### Treatment comparison-RDK

**Figure 1:**
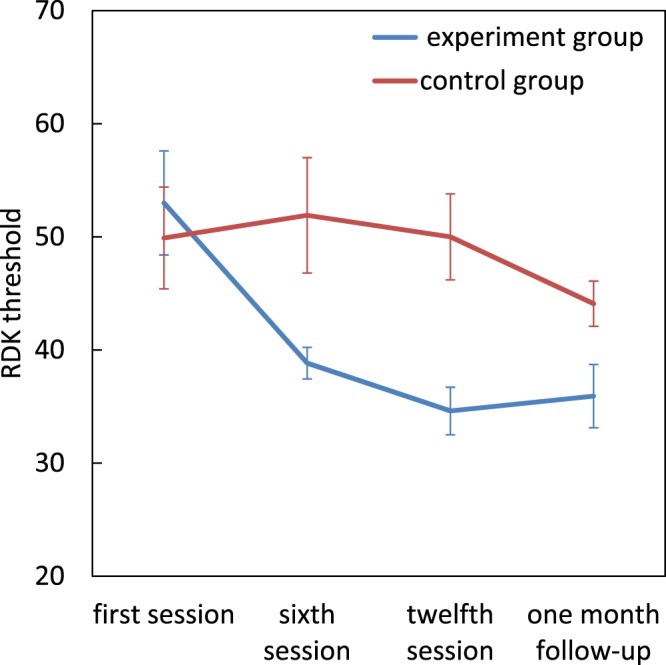
Effect of magnocellular training on RDK threshold. Error bars represent SEM.

There were significant main effects for group (*F*[1, 22] = 6.20, *p* = 0.02, η^2^_p_ = 0.22) and the assessment session (*F*[3, 66] = 5.55, *p* = 0.002, η^2^_p_ = 0.20) and also significant group × assessment session interaction (*F*[3, 66] = 3.81, *p* = 0.01, η^2^_p_ = 0.14). There were no significant differences between the two groups at the first session, but the dyslexics in the experimental group had lower thresholds by the sixth session (t(22) = −2.64, *p* = 0.015), twelfth session (t(22) = −3.63, *p* = 0.001) and also at the 1 month follow-up (t(22) = −2.250, *p* = 0.035) compared to the control group. Pairwise comparisons within the experimental group, showed a significant reduction in RDK threshold from first session to sixth session (mean difference = 14.154, *p* = 0.004), twelfth session (mean difference = 18.385, *p* = 0.002) and at follow-up (mean difference = 17.077, *p* = 0.013), but there were no significant changes for the control group (*p* > 0.05) (Fig. 1).

### Saccadic eye movements

**Figure 2:**
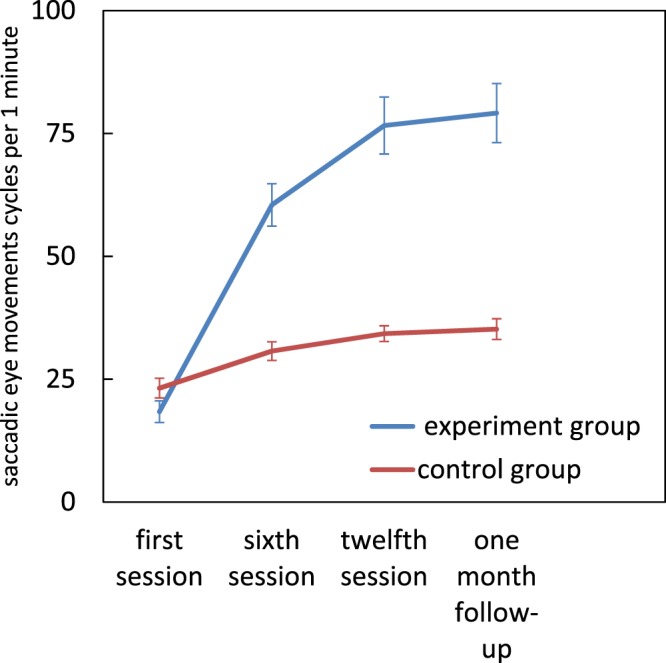
Effect of magnocellular training on saccadic eye movements. Error bars represent SEM.

There were significant main effects for group (*F*[1, 22] = 32.43, *p* < 0.001, η^2^_p_ = 0.59) and assessment session (*F*[1.73, 38.21] = 111.11, *p* < 0.001, η^2^_p_ = 0.83), as well as a significant group × assessment session interaction (*F*[1.73, 38.21] = 50/68, *p* < 0.001, η^2^_p_ = 0.69). There were no significant differences between the two groups at the first session, but the dyslexics in the experimental group had higher saccadic eye movement scores by the sixth session (t(22) = 5.88, *p* = 0.0001), the twelfth session (t(22) = 6.415, *p* = 0.0001) and also at follow-up (t(22) = 6.409, *p* = 0.0001) compared to the control group. Pairwise comparisons within the experimental group, showed a significant improvement from the first to the sixth session (mean difference = −42.077, *p* = 0.0001), the twelfth session (mean difference = −58.231, *p* = 0.0001) and at follow-up (mean difference = −60.769, *p* = 0.0001) (Fig. 2).

### Reading accuracy

**Figure 3:**
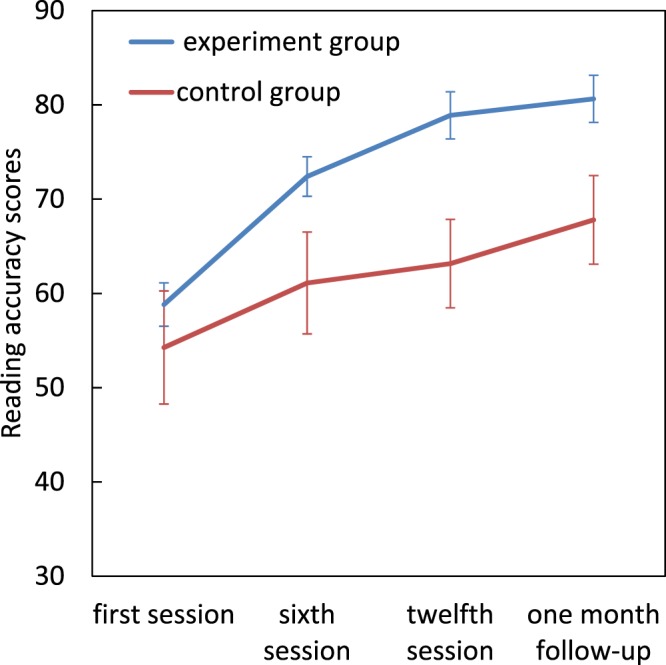
Effect of magnocellular training on reading accuracy scores. Error bars represent SEM.

Repeated measures ANOVA revealed significant main effects of group (*F*[1, 22] = 4.36, *p* = 0.048, η^2^_p_ = 0.16) and assessment session (*F*[1.62, 35.66] = 88.99, *p* < 0.001, η^2^_p_ = 0.80) as well as a significant Group × assessment session interaction (*F*[1.62, 35.66] = 8.42, *p* = 0.002, η^2^_p_ = 0.27). There were no significant differences between the two groups in the first session, but the dyslexics in the experimental group had higher reading accuracy scores by the twelfth session (t(22) = 3.031, *p* = 0.006) and also at follow-up (t(22) = 2.51, *p* = 0.020) compared to the control group. Pairwise comparisons within the experimental group, showed a significant improvement from first session to sixth session (mean difference = −13.58, *p* = 0.0001), twelfth session (mean difference = −20.07, *p* = 0.0001) and at the 1 month follow-up (mean difference = −21.822, *p* = 0.0001) (Fig. 3).

### Reading Comprehension

**Figure 4:**
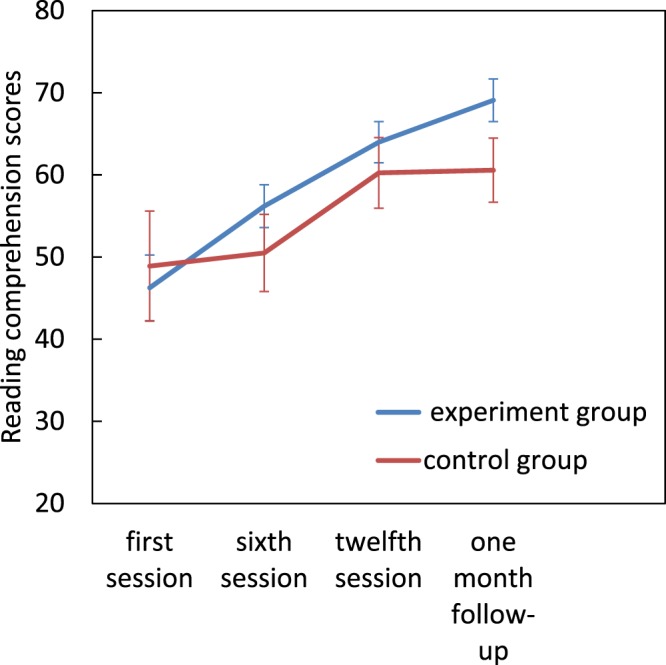
Effect of magnocellular training on reading comprehension scores. Error bars represent SEM.

For comprehension there was a significant main effect of the assessment session (*F*[1.57, 34.64] = 15.16, *p* < 0.001, η^2^_p_ = 0.40), but there was no significant effect of group (*F*[1, 22] = 0.732, *p* = 0.40), nor a significant group × assessment session interaction (*F*[1.57, 34.64] = 1.34, *p* = 0.26). Pairwise comparison revealed a significant improvement in comprehension after treatment from the first to the sixth session (mean difference = 5.774, *p* = 0.044) to twelfth session (mean difference = 14.541, *p* = 0.001) and to follow-up (mean difference = 17.252, *p* < 0.001) (Fig. 4).

An independent samples t test showed a significant difference between the experimental group and the control group’s text comprehension only during the follow-up session (t(22) = 2.5, *p* = 0.02).

### Reading errors

#### Visual errors

Repeated measures ANOVA revealed no significant main effects for group (*F*[1, 22] = 1.63, *p* = 0.21), for assessment session (*F*[1.84, 40.64] = 2.79, *p* = 0.07) nor a significant group × assessment session interaction (*F*[1.84, 40.64] = 2.32, *p* = 0.115) for visual errors.

**Figure 5:**
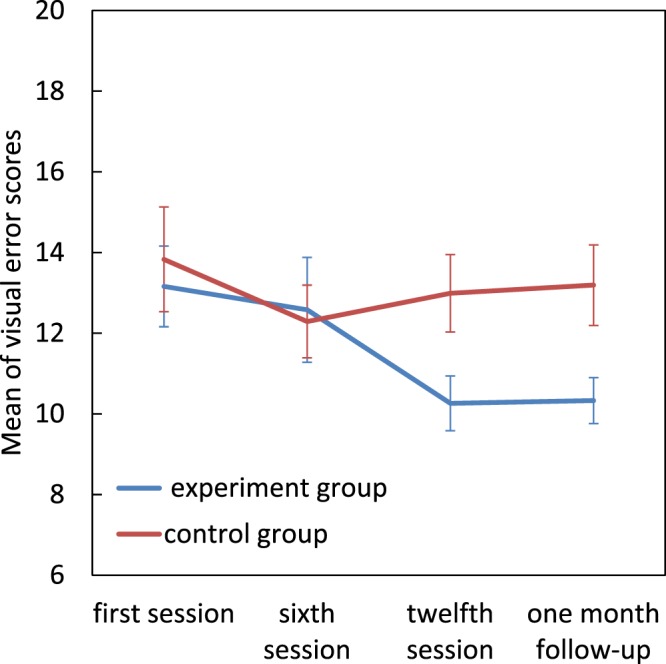
Effect of magnocellular training on visual errors. Error bars represent SEM.

An independent sample t-test showed lower visual errors in the experimental group compared to the control group only at the twelfth session and one month follow-up (t(22) = −2. 36, p = 0.027; t(22) = −2.41, p = 0.024) (Fig. 5).

#### Phonological errors

**Figure 6:**
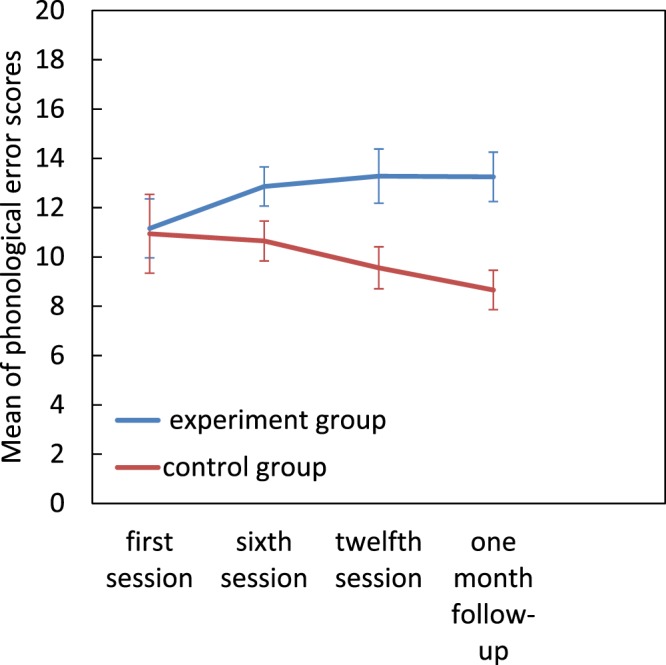
Effect of magnocellular training on phonological errors. Error bars represent SEM.

For the phonological errors there was a significant group × assessment session interaction (*F*[3, 66] = 4.49, *p* = 0.006, η^2^_p_ = 0.1. Pairwise comparisons revealed a significant increase in phonological errors between the first and final assessments in the experimental group, but no other significant effects (*p* > 0.05) (Fig. 6).

There were no significant observations for pragmatic errors.

## Discussion

Since the magnocellular pathway plays such a crucial role for focusing visual attention and letter decoding^38^, a deficit in this pathway is likely to be an important cause of reading difficulties^39^. Our study has confirmed that visual magnocellular training in primary school children with reading difficulties improves their detection of coherent motion as well as their control of saccadic eye movements and that these improvements are accompanied by increased reading accuracy and reduced visual errors, whereas there were no significant changes in the control group. While both groups showed improvements in text comprehension, the difference between the control group and the experimental group became significant only in the follow-up session. These results are consistent with many studies which have found that sensitivity to motion detection and also eye movement control can be increased with repeated exposure and practice, and that this is accompanied by reading improvements^11,32–36^. Although Conlon, Sanders and Wright (2009), reported that the motion detection deficit is persistent in dyslexics and did not benefit from practice^40^, they had already given their participants 35–40 trials at their initial assessment which meant that they had already had a great deal of practice at the task.

Cornelissen *et al*. (1998) studied the relationship between coherent motion detection, letter decoding ability and visual reading errors^22^. They and others showed that deficits in the magnocellular pathway were particularly associated with visual reading errors like omissions and additions^22,41,42^. It was not surprising therefore, that in our study (Fig. 5) we showed a significant reduction in the experimental group’s visual reading errors while for the control group these errors remained more or less unchanged. The improvement in motion detection (RDK scores) began by the sixth session of training whereas the decrease in visual reading errors occurred only at the end of the intervention, as obviously would be expected if the improvement in magnocellular function is what causes the decrease in reading errors later, rather than vice versa.

Somewhat unexpectedly the number of phonological errors increased in the experimental but decreased in the control group. This finding is in contrast to Lawton (2016) and Lawton and Shelley Tremblay (2017) who reported decreases in phonological errors for both dyslexic and typically developing readers who did the training. They concluded that reduction in phonological errors is the result of improvement in low level and high-level functioning in the dorsal stream. However, these contrasting findings may be explained by the dual route model of reading. According to this model, whole words are automatically identified by their visual form if they are familiar via the lexical route, and so reading is faster and more accurate. But for unfamiliar words, the word has to be broken down into its constituent letters and corresponding phonemes; this is the sub- lexical route^43^. Impairment in either of these routes will result in a characteristic pattern of reading difficulties^44^. Siegel (1993) found that students who had deficits in phonological skills (the sub-lexical route) tried to use the lexical route instead, in order to compensate^45^. In our study, participants probably tried to use the sub-lexical because of their problems with the lexical route. After intervention however, participants in the experimental group were able to use the lexical route well. Hence in the reading test, they began to rely on the visual form of the words, so their phonological errors increased. But as Fig. 6 shows, by the 1 month follow up their phonological error rate had plateaued, and it would probably have begun to decrease later as the children reached a correct balance in the use of these two routes.

As discussed earlier, other studies have used other methods for magnocellular intervention. For example, Lawton (2016) and Lawton and Shelley-Tremblay (2017) had shown that figure-ground movement discrimination training resulted in improvements in reading fluency, visual timing deficits, attention, phonological processing and also working memory. Our results showed that the intervention method used in this study improved magnocellular function, saccadic eye movement and also reading accuracy and reduced visual errors. Future studies are required to compare different intervention methods in Persian speaking students. Probably the most effective intervention will combine the different methods.

This study has some clear limitations. First, the selection of participants with reading difficulties was mainly based on qualitative teacher evaluations and their performance on APRA and RDK. This did not select children whose reading difficulties were solely the result of a visual magnocellular pathway deficit, with no other problems. Hence some of their reading difficulties might have been due to auditory or other causes. Secondly, in all of our assessment sessions we used the same texts to assess the children’s reading and comprehension skills. Hence through repetition the children could simply have learnt the words and thus do better in the tests. This can partially explain the lack of significant group*session interaction in the comprehension test. However, this should have affected both groups equally. Instead the controls’ performance fell far behind that of the experimental group. Third, although the participants were blind to which treatment might be effective, due to some practical limitations it was not possible to blind the experimenter. Thus, they could have influenced the assessments in favor of the treatment. Fourth, the effect sizes acquired in our sample were mostly medium^46^, which suggest that there is a chance of missing significant differences in our analyses. Hence, future studies may benefit from a bigger sample and inclusion of a control group of normal readers.

## Conclusion

Successful acquisition of reading skills depends crucially on the function of the visual magnocellular system. Here we show that improving its performance by magnocellular training strategies benefitted a sample of children with visual problems and dyslexia. Unfortunately, the importance of magnocellular visual processing is still not well recognized in the majority of learning disabilities clinics. Hopefully these results will encourage clinicians to pay more attention to these visual problems. In addition, since magnocellular sensitivity in infancy predicts reading abilities later in school, early magnocellular screening may be an easy and useful way to identify children at risk of dyslexia.

## Methods

### Participants

The study was conducted on 24 male primary school students with reading difficulties (mean age: 9.76 years, and range: 9–11 years). Inclusion criteria were:

1. a reading score below 71 on APRA. This cut off was established by Bakhshalizade (2012)^20^.
2. a threshold score above 34 in a random dot kinematogram (RDK) test; this score was 2 SDs above the reported mean for non-dyslexic students in a similar population^20^.
3. absence of any neurological conditions or sensory deficits, including visual.
4. normal IQ with no history of either clinical or educational interventions during the course of their education so far.

All the children agreed voluntarily to join the study, and their parents provided written informed consent under a protocol that was approved by the Shahid Beheshti University and all methods were performed in accordance with the relevant guidelines.

### Study design

Randomly selected, 13 children were allocated to the experimental and 11 to the control group. These groups were well matched on age, sex, educational level and IQ. The experimental group received 12 magnocellular training sessions lasting 30–40 minutes over a period of 6 weeks. The control group played a video game on windows 7 which was used to eliminate the effect of working with a computer and also the interaction between examiner and students. This game was designed as a bakery and was about cooking cakes and cookies. The assessments were repeated 4 times: just prior to the first intervention session, after the 6^th^ session, after the 12^th^ session and at a follow-up appointment one month later.

## Assessments

### Persian Reading Ability (APRA)

To assess their reading ability the APRA reading and comprehension test was used^2^. Reading errors in this test fell into three categories:

1. Visual errors: Addition (extra words or letters are inserted into text), Omission (words are fully or partially omitted) and Reversal (word letters are reversed).
2. Phonological errors: Mispronunciations (words wrongly pronounced or distorted); Substitutions (incorrect real words with the same initial phoneme used instead of the target words); Fragmentation (words broken into components and then combined to read).
3. Pragmatic errors: Refusals (participant pauses for 5–7 seconds with no effort to read) and Repetition (participant reread the whole word)^2^.

APRA has been used in many different studies and settings and has proved to be a valid and reliable measure for reading words, passages and for testing comprehension^47–49^.

**Figure 7:**
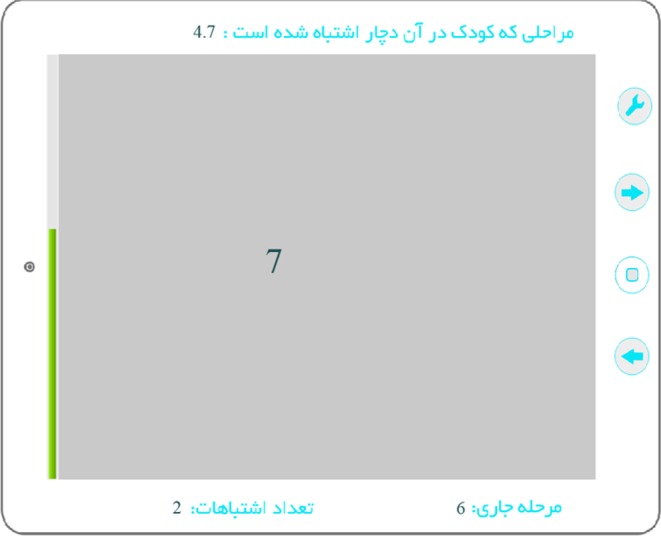
Is snapshot of a trial presentation. Top of the figure shows the level in which the participant has made errors (4.7), the right bottom shows the current level (6) and the left bottom is total errors (2).

### Random Dot Kinematogram (RDK)

A two panel RDK was used to assess dorsal route/magnocellular function^4^. Two sets of moving dots appeared in two panels side by side. One set contained a proportion of dots which moved coherently to the left or right together with noise dots that moved in random directions. The other set contained only noise dots. The participants were asked to identify on which side a cloud of dots appeared to be moving together (coherently). The proportion of dots that moved coherently was adjusted in a staircase so that each correct answer decreased the proportion of dots moving coherently by 1 dB and each wrong answer increased it by 3 dB. The test went on until the proportion of coherent dots had reversed 8 times. Threshold was defined as the proportion of dots which had to move together for the participant to see the coherent motion correctly on 75% of trials.

### Saccadic eye movements

In order to evaluate saccadic eye movement control, two targets were placed at each end of a 30-cm ruler, held horizontally 20 cm away from the participant’s eyes, head fixed viewing the middle of the ruler in line with the nose. Then, he was asked to move his gaze from one target to the other as quickly as possible. Each target had to be clearly seen after each saccade. Eye movements were monitored and recorded during the task. A saccade to the left followed by one to the right was called *a cycle*. The number of cycles made per minute was recorded^36,50^.

### IQ test

Raven’s Progressive Matrices.

### Magnocellular based visual motion training

The children performed the following computerized tasks on a 17- inch personal computer screen, (Windows 7), located 50 cm away. They used a chin rest for the Digit and Dot Counting tasks.

#### Saccadic eye movement training

Prior to training, how to perform the saccadic eye movements was explained and practiced with each child. The saccadic eye movement training was similar to the assessment. For this training, we used a previously developed task which was based on repeated alternating visual focus practice^36^. Two identical targets were placed at each end of a 30-cm ruler and the ruler was kept horizontally 20 cm away from the participant’s eyes, head fixed with the middle of the ruler in line with the nose. The experimenter asked the subject to move his gaze from one target to the other alternatively as quickly as possible. Similar to the assessment test, a saccade to the left followed by one to the right was called *a cycle*. Each correct cycle was counted out loud to provide feedback so that the child learned to recognize when his saccades were correct. Eye movements were monitored and recorded by the examiner during the task. Following Sabet (2013)^36^, each intervention session then began with 20-minutes saccadic eye movement training divided into 10 one minute trials with a 1 minute break between trials. During each trial, the distance between the two targets on the ruler was gradually decreased. In order to improve up-down eye movement control, the ruler was placed vertically^51^ during some trials.

#### RDK (version modified for training)

This was similar to the RDK test, but with a feedback noise to indicate when choices were incorrect. The task was stopped after total of 4 wrong choices.

Two other tasks were designed based on Leong *et al*. (2014) and Dodic *et al*. (2016)^34,35^:

#### Digit Counting

This task had 10 progressive levels. At the beginning of each level, a digit between 0–9, selected randomly, was presented on the right side of the screen (printed in black with a font in a grey background, for an example screenshot see Fig. 7). After a few milliseconds (depending on the level) the number disappeared and was replaced by another random number on the left side of the previous number (the reading and writing direction in Persian is right to left). As the level went up, both digit presentation time and font size were decreased. At the first level, presentation time was 560 ms, and it decreased to 190 ms for the 10^th^ level. The children were asked to choose a favorite number to begin each trial and then report how many times that number had been presented. A feedback (happy or sad emoji face) was provided after each answer. After each correct answer the level was increased. After each wrong answer, the program regressed a level back and after four mistakes, it was stopped altogether.

#### Dot Counting Task

A variable number of black dots (all the same size) were presented sequentially within a virtual square on a grey background (each dot disappeared after presentation). Participants were asked to trace visually and count the number of dots presented at a given level. Auditory feedback was provided after each response. At the first level, 7–9 dots were presented in 9 seconds. The children proceeded to the next level if they made correct responses in 80% of trials at the current level. In each level, the number of presented dots increased, and their interval time decreased by 25% compared to the previous level.

#### Between-session training

Between the sessions at our lab the children practiced the task at home. Parents were instructed how to perform the tasks and given a homework chart with full instructions. They could also use a free telephone line for support. At the next lab session, children were rewarded if their homework charts had been completed.

#### Visual accommodation training

Participants were asked to read a short story printed in two fonts. The large font size (36), was placed 4 m from the child, whilst the small font size (14) was placed 30 cm away. With the assistance of their parents the children were first asked to read a line from the small font print, then one from the large font. This practice was carried out twice a day, for 10 minutes each.

The second task to perform at home was a printed version of Digit Counting Task. A notebook was provided with 7 pages of rows of random digits between 0 to 9. Each page varied in the number of digits as well font size. Participants were instructed to read the digits on each page as loudly and as quickly as possible with no mistakes. If they made a mistake or omission, they had to go back one page. During the practice, the children’s heads were held still by their parents. This task was also done twice a day, for 10 minutes.

### Statistical analysis

A repeated measures ANOVA was carried out by group (control vs. experimental) as the between-subjects factor and assessment time (pre-test, mid-test, post-test, follow-up) as the within-subject factor. Greenhouse-Geisser corrections were performed if the Mauchuly’s assumption had not been violated. A set of t-tests and Man-Whitney U tests were also carried out.

## Supplementary information

Dataset 1

## Data Availability

All data associated with this manuscript is available at: https://www.nature.com/.
