# Supplementary material for: Magnocellular Based Visual Motion Training Improves Reading in Persian
Source: Sci Rep. 2019 Feb 4;9:1142. doi: 10.1038/s41598-018-37753-7 (PMC6361887; doi:10.1038/s41598-018-37753-7)
Supplement: Supplementary file 1 — Dataset 1 [file 41598_2018_37753_MOESM1_ESM.zip › Magnocellular based visual motion training/RDK/doc/index.html]

Random Dot Testing
